# Impact of Thermal Variations on the Fatigue and Fracture of Bi-Material Interfaces (Polyimide–EMC, Polyimide–SiO_2_, and Silicon–EMC) Found in Microchips

**DOI:** 10.3390/polym17040520

**Published:** 2025-02-17

**Authors:** Pedro F. C. Videira, Renato A. Ferreira, Payam Maleki, Alireza Akhavan-Safar, Ricardo J. C. Carbas, Eduardo A. S. Marques, Bala Karunamurthy, Lucas F. M. da Silva

**Affiliations:** 1 Department of Mechanical Engineering, Faculty of Engineering (FEUP), University of Porto, Dr. Roberto Frias Street, 4200-465 Porto, Portugallucas@fe.up.pt (L.F.M.d.S.); 2 Institute of Science and Innovation in Mechanical and Industrial Engineering (INEGI), Dr. Roberto Frias Street, 4200-465 Porto, Portugal; 3 Infineon Technologies Austria AG, Siemensstrasse 2, 9500 Villach, Austria

**Keywords:** bi-material interface, fracture, fatigue, delamination, polyimide, epoxy molding compound, silicon, semiconductors

## Abstract

As the trend towards the densification of integrated circuit (IC) devices continues, the complexity of interfaces involving dissimilar materials and thermo-mechanical interactions has increased. Highly integrated systems in packages now comprise numerous thin layers made from various materials. The interfaces between these different materials represent a vulnerable point in ICs due to imperfect adhesion and stress concentrations caused by mismatches in thermo-mechanical properties such as Young’s modulus, coefficients of thermal expansion (CTE), and hygro-swelling-induced expansion. This study investigates the impact of thermal variations on the fracture behavior of three bi-material interfaces used in semiconductor packaging: epoxy molding compound–silicon (EMC–Si), silicon oxide–polyimide (SiO_2_–PI), and PI–EMC. Using double cantilever beam (DCB) tests, we analyzed these interfaces under mode I loading at three temperatures: −20 °C, 23 °C, and 100 °C, under both quasi-static and cyclic loading conditions. This provided a comprehensive analysis of the thermal effects across all temperature ranges in microelectronics. The results show that temperature significantly alters the failure mechanism. For SiO_2_–PI, the weakest point shifts from silicon at low temperatures to the interface at higher temperatures due to thermal stress redistribution. Additionally, the fracture energy of the EMC–Si interface was found to be highly temperature-dependent, with values ranging from 0.136 N/mm at low temperatures to 0.38 N/mm at high temperatures. SiO_2_–PI’s fracture energy at high temperature was 42% less than that of EMC–Si. The PI–EMC interface exhibited nearly double the crack growth rate compared to EMC–Si. The findings of this study provide valuable insights into the fracture behavior of bi-material interfaces, offering practical applications for improving the reliability and design of semiconductor devices, especially in chip packaging.

## 1. Introduction

Chip packaging engineering has evolved significantly to meet the increasing performance demands of integrated circuits (ICs) and microelectromechanical systems (MEMSs). Silicon wafers, produced using the Czochralski method [1], serve as the foundational material for circuits. To safeguard these circuits and facilitate electrical connections, layers or components predominantly composed of metals, ceramics, and polymers are affixed to the wafer [2,3]. This evolution has led to the categorization of chip packaging into two main technologies: non-advanced packaging (e.g., wire bonding and flip chip) and advanced packaging (e.g., wafer-level packaging and 2.5D and 3D devices) [3].

However, these packaging technologies face significant challenges, particularly with problems such as delamination and popcorning [4], which occur due to thermal expansion coefficient mismatch, especially during solder reflow processes [5]. Therefore, it is essential to guarantee and analyze the strength of bi-material interfaces in such components to ensure a reliable product. Linear elastic fracture mechanics allow for studying and understanding the properties of bi-material interfaces in the three modes of fracture (opening, in-plane shear, and out-of-plane shear). In recent years, several setups have been used/proposed to characterize bi-material interfacial properties. The most considered methods include double cantilever beam (DCB) [6,7], four-point bending (4PB) [8,9], mixed-mode bending (MMB) [10], button shear (BS) [11,12], and peel tests [13]. These studies are complemented with fatigue analysis, where fatigue crack growth behavior can be analyzed and product failure can be predicted using the Paris–Erdogan law. Interfaces such as EMC–PCB [14], EMC–Cu [15,16], and PI–EMC [17] have been investigated and compared [18] under different temperatures, leading to changes in interfacial properties. In mode I, DCB tests have been compared for these interfaces at room temperature, with PI–EMC having the lowest fracture energy at 37 J/m^2^. Additionally, at high temperatures, EMC–PCB and Si–Underfill showed a reduction in their fracture energies. While this reduction was initially linear, a rapid reduction in fracture energy was reported upon passing the glass transition temperature ($T_{g}$) of the EMC. This highlights the necessity of studying these interfaces in more detail at high temperatures, as well as at low temperatures since no studies were found on the effect of low temperatures (below zero) on the fracture and fatigue response of such interfaces. In mode II, similar tests have been conducted using four-point bending configurations. Different results have been reported for such cases. While interfaces such as EMC–Cu(oxide) and EMC–PCB showed reductions in their fracture energy at higher temperatures, the opposite occurred for EMC–Cu, with the fracture energy increasing with temperatures up to the $T_{g}$ of EMC, where it then decreases. This confirms that although a decrease in temperature generally reduces the fracture energy, this effect may not be observed for all interfaces. Sankarasubramanian et al. [19] utilized a DCB testing system along with an environmental control chamber to assess the adhesion energy between the underfill and silicon layers in a flip chip microelectronic package across various temperatures. The findings indicated that the adhesion strength diminished as the temperature rose above the glass transition temperature ($T_{g}$) of the underfill. Yeh et al. [20] used four-point bending (FPB) tests to examine the interfacial bonding strength between the underfill material and the polyimide (PI) lamination layers in advanced fan-out packages, assessing the effects of varying temperatures and the different treatments of the PI surface. Their findings revealed that as the temperature increased, the critical strain energy release rate ($G_{C}$) decreased, while the interfacial strength improved after the surface treatment of the PI. The interfaces of EMC–LF (leadframe) are notably weak when the environmental temperature exceeds the glass transition temperature of the EMC, placing it in a rubbery state with a significantly reduced Young’s modulus. Studies indicate that even near the glass transition temperature ($T_{g}$ = 120 °C), the interface’s toughness is substantially diminished, being 60–70% lower than at an ambient temperature [21].

For shear testing, a British standard was used to compare the thermal effects on PI–EMC, EMC–-Ni, Si–UF, and Si_3_N_4_. It was found that shear strength decreased with temperature due to the loss of the mechanical properties of EMC and the epoxy contained in the underfill parts [22,23]. Despite this, most of these studies only consider high-temperature applications (60–300 °C) and neglect the effects of low temperatures. The only study conducted at low temperatures [15] is dedicated to EMC–Cu, with tests performed at −50 °C. Compared to results at room temperature, the fracture energy was slightly increased at low temperatures. It is also important to note that there is a scarcity of studies on the influence of temperature on the fatigue delamination analysis of bi-material interfaces in microchips. In a study on fatigue delamination in EMC–LF, Poshtan et al. [24] observed that, although some molding compound particles remained on the leadframe surface, an interfacial fracture was the predominant failure mode. Samet et al. [25] analyzed the fatigue fracture of the EMC–Cu (copper) interface in a DCB setup where ∆*G* (range of strain energy release rate) was used in relation to the Paris law. The *m* (the slope of the Paris law curve) was found to be around 10. The samples lasted approximately 2000 cycles before failure. The same Paris law relationship was used in another study [26] where the SiN–PI interface was investigated under cyclic loading. The *m* exponent was determined as 3.2 for this interface. In this study, experiments were conducted with a constant load amplitude in a triangular waveform.

It is known that temperature [27,28], or even cyclic variations in temperature, affects the performance of joints [29] and is an important factor to consider regarding the delamination between materials used in chip applications [30]. Not only do thermal stresses contribute to delamination, but changes in material properties, especially for materials with glass transition temperatures ($T_{g}$) lower than or near the testing temperatures, also play a significant role [22,23]. Furthermore, the dependence of these material properties on temperature changes significantly influences the interfacial fracture behavior [31]. One interface that has not been thoroughly studied for mode I characterization under different temperature conditions is EMC–silicon. Over the years, EMC–Si interface delamination has been regarded as a challenge due to the brittle behavior of silicon. Schlottig [32] was the first to delaminate these two materials. For this, a mixed-mode chisel setup was developed around the specimen geometry to quantify the fracture energy for the first time [33,34]. Morais et al. [35] were the first to delaminate these two materials in pure mode I condition.

This review highlights a lack of understanding of the full scope of the effects of temperature across all temperature ranges in microelectronics. Furthermore, while fatigue analysis has been conducted on interfaces such as EMC–Cu and SiN–PI, there is a lack of studies focusing on the fatigue delamination of the EMC–Si, PI–EMC, and SiO_2_–PI interfaces under different temperature conditions. This oversight is particularly concerning given the different behavior of these materials and the challenges associated with delaminating these materials.

This work aims to analyze the effect of temperature on different types of interfaces in microchips, including epoxy molding compound–silicon (EMC–Si), silicon oxide–polyimide (SiO_2_–PI), and PI–EMC. DCB samples were manufactured and loaded under pure mode I condition at different temperatures of −20 °C, 23 °C, and 100 °C. The samples underwent both quasi-static and cyclic loading conditions. Results from static and fatigue testing are compared with data reported in the literature for other interface types.

## 2. Experimental

### 2.1. Materials

Specimens were extracted from a wafer manufactured using the wafer-level packaging (WLP) manufacturing procedure. The samples, each measuring 100 mm × 25 mm, were cut from a 200 mm wafer using a diamond saw.

**Figure 1:**
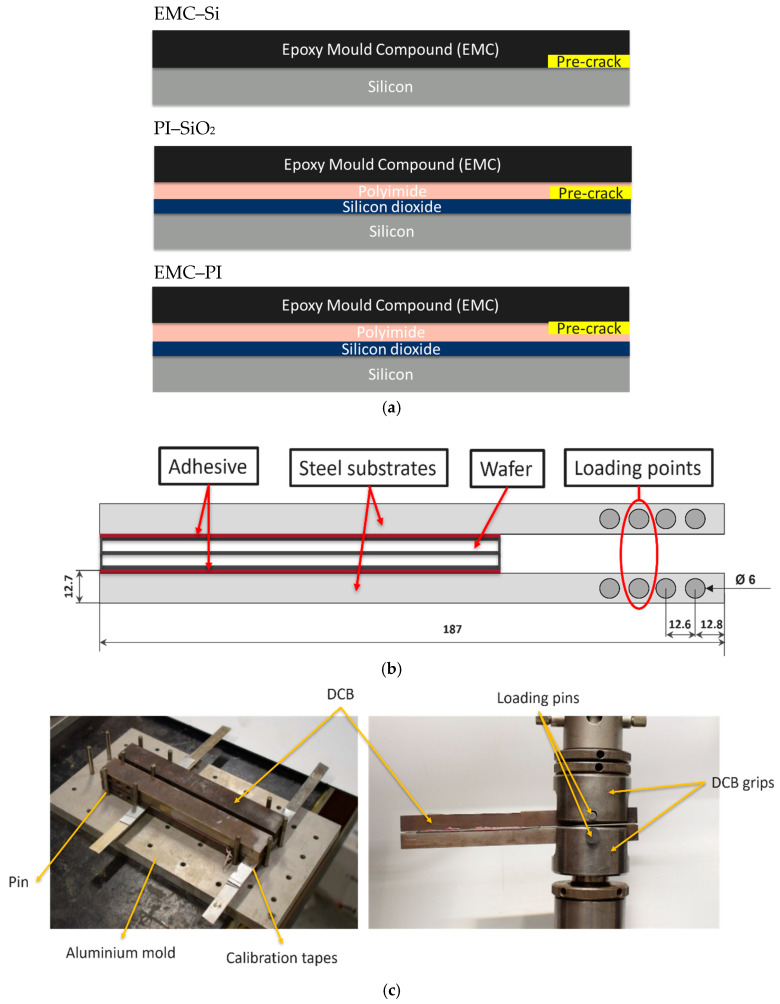
(**a**) The stacking sequence and arrangement of layers in three different specimens, (**b**) the DCB specimen, and (**c**) DCB manufacture and mode I fracture test set-up (dimensions in mm). The schemes shown in this figure are not to scale.

The interfaces under study are composed of multilayers of distinct materials. For the EMC–Si interface under study, there are no additional layers on the wafer’s specimen. However, for the PI–EMC interface, there are layers of Si and silicon dioxide (SiO_2_), while for the SiO_2_–PI interface, the specimen also includes a layer of EMC and silicon. Between the two interfaces, a thin pre-crack was created on the initial 20 mm of the wafer to serve as a pre-crack. Figure 1a shows the exact configuration and stacking sequence of the layers. Table 1 provides information on the properties of the materials used in this study.

### 2.2. Joint Geometry and Manufacturing

The dimensions and geometry of the DCB joint in this study deviate slightly from the ASTM D3433-99 standard [39] due to the availability of PM300 steel substrates. Figure 1b illustrates the dimensions of the joint and the position of the wafer in the DCB setup.

In our study, the DCB joints involve two PM300 substrates with the pertinent bi-material interface specimen positioned between them as shown in Figure 1b. To apply the load, the vertical pair of holes in the steel bars is utilized. These adjustments in geometry and dimensions are crucial to ensure alignment with the unique properties of the PM300 steel substrates, enabling accurate and pertinent experimental outcomes (Figure 1c). Due to the elastic mismatch between the EMC and the silicon, achieving a fully mode I fracture may be challenging. However, supporting the bi-material specimen with symmetrical thick and stiff substrates allows us to mitigate the elastic mismatch and consider only the modulus of the steel supports for fracture energy calculation. This enables us to treat the problem as a pure mode I scenario.

In order to support the specimens using steel beams, it is necessary to bond the beams to the wafer using adhesives. Therefore, the initial step in sample manufacturing involves selecting a suitable adhesive for this application. Due to the high- and low-temperature requirements, two different adhesives were selected: 3M Scotch Weld 163-2K 0.06 (manufactured by 3M, based in Saint Paul, Minnesota, United States), a structural epoxy film adhesive, was selected for low-temperature applications due to its capability to provide strong bonding in colder environments, and DELO^®^ MONOPOX AD286 (manufactured by DELO, located in Windach, Germany), an epoxy paste, for high-temperature scenarios as it is designed to withstand extreme thermal stress without losing adhesion properties. It should be noted that the Delo epoxy adhesive was applied to the surface using a spatula.

The PM300 bars’ surfaces were sandblasted to clean them and improve the adhesion between the adhesive and steel bars. Spare sand and dust were then removed from the substrates using compressed air and acetone degreasing. The preparation of wafer surfaces involved manual abrasion with 600-grit sandpaper at ±45° angles. Subsequently, the surfaces underwent degreasing with acetone and a 6 s plasma treatment using the Arcotec PG051 device (manufactured by Arcotec GmbH, based in Mönchengladbach, Germany). These surface treatments enhanced the surface energy [40] of the wafers, promoting better adhesion to the steel substrates. The 3M Scotch Weld 163-2K 0.06 tapes were trimmed with a 3.5 mm smaller offset compared to the steel substrates. This allowed the adhesive to flow and bond to the entire surface while preventing bonding to the side interfaces. Various tape sizes were tested to optimize adhesive flow control. To ensure precise adhesive flow and thickness, layers of calibration tape, each measuring 1.7 mm thick, were carefully applied. This meticulous process establishes the groundwork for effective bonding and reliable experimental outcomes in subsequent manufacturing phases.

After completing these steps, the specimens are ready for curing. The curing cycles were tailored for the two different adhesives. For 3M Scotch Weld 163-2K 0.06, the adhesive was cured at 120 °C for 90 min. For DELO® MONOPOX AD286, the adhesive was cured at room temperature for 24 h. It is important to note that curing the adhesive introduces residual stresses, not only to the adhesive itself but also to the wafer [41]. Elevated curing temperatures can lead to differential thermal expansion between the adhesive and the wafer material. If the adhesive expands or contracts at a different rate compared to the wafer during cooling, it can induce residual stresses that may affect the structural integrity and performance of the wafer.

### 2.3. Testing Conditions

The objective of this study is to understand the behavior of EMC–Si interfaces when subjected to static and fatigue loads at high, room, and low temperatures. The rationale for selecting these temperatures is that they represent a range of conditions that the material encounters during service, reflecting the real-world application of the product. The testing conditions are shown in Table 2. Results for PI–EMC and EMC–Si interfaces at room temperature were considered from a previous study [35]. During testing of these joints, three types of failure mechanisms were observed: silicon failure, mixed failure (where the crack is kinked between two or more materials), and interfacial failure. Although all conditions (fatigue and static fracture at all three temperatures and for all three types of interfaces shown in Figure 1) were tested, this work will present only the results pertaining to interfacial delamination since the goal is to characterize the interfaces. However, for future work, other failure mechanisms will be tested and analyzed.

It is important to note that the tested specimens exhibit residual stresses and were subjected to loading during the manufacturing process, as evidenced by the warpage of the specimens. The failure mechanisms observed during testing are influenced by the manufacturing history that the specimens experience during wafer manufacturing.

Testing was performed using two different machines. An INSTRON 3367 testing machine (manufactured by Instron, headquartered in Norwood, MA, USA) with a load cell capable of measuring up to 30 kN was used for the quasi-static mode I DCB tests, under room temperature conditions. The displacement rate for these tests was 0.2 mm/min. For the low- and high-temperature requirements, an Intrusion 3119-600 chamber was used to control the temperature during the test. An INSTRON 8801 testing framework (manufactured by Instron, which is headquartered in Norwood, MA, USA) was selected for fatigue tests, featuring a load cell rated at ±100 kN. A basic sine wave, which can be controlled and described by displacement, load, or stress, was used to define the fatigue loading conditions. The fatigue tests were load-controlled, requiring only three parameters: frequency, loading ratio (R), and maximum fatigue load. The frequency was set to 1 Hz and the R ratio was 0.33 for the fatigue tests.

### 2.4. Data Reduction Approach

Precisely measuring crack length during fracture testing presents challenges, especially under cyclic loading. The compliance-based beam method (CBBM) addresses this issue. With this data reduction approach based on linear elastic fracture mechanics, there is no need to monitor the crack length during testing. Instead, an equivalent crack length is utilized. The method derives its estimates solely from the specimen’s compliance (*C*).

From Castigliano’s theory, the displacement, δ, can be written as [42]:

(1) $$\delta =\frac{\partial U}{\partial P}=\frac{8aP^{3}}{EBh^{3}}+\frac{12Pa}{5BhG}$$

where *a* is the crack length, *P* the load applied, *h* and *B* are the substrate’s thickness and width, respectively, and *G* is the flexural modulus. The equivalent crack length, $a_{eq}$, can be obtained from the compliance of the test specimen using the following equation:

(2) $$C=\frac{\delta }{P}=\frac{8{\left(a_{eq}\right)}^{3}}{Bh^{3}E_{f}}+\frac{12a_{eq}}{5GBh}$$

where *E_f_* is the equivalent flexural modulus. Pure mode I fracture energy, *G_I_*, is calculated as follows [42]:

(3) $$G_{I}=\frac{6P^{2}}{b^{2}h}\left(\frac{2{\left(a_{eq}\right)}^{2}}{h^{2}E_{f}}+\frac{1}{5G}\right)$$

## 3. Results and Discussion

### 3.1. Static

The room temperature static strength of these bi-material interfaces has already been presented and discussed in a previous study [35]. However, it is crucial to understand the evolution and compare the differences in results by changing the loading conditions. Therefore, some results will be contextualized in this paper. This work will further investigate and compare those results obtained at different testing temperatures.

#### 3.1.1. Room Temperature Results

**Figure 2:**
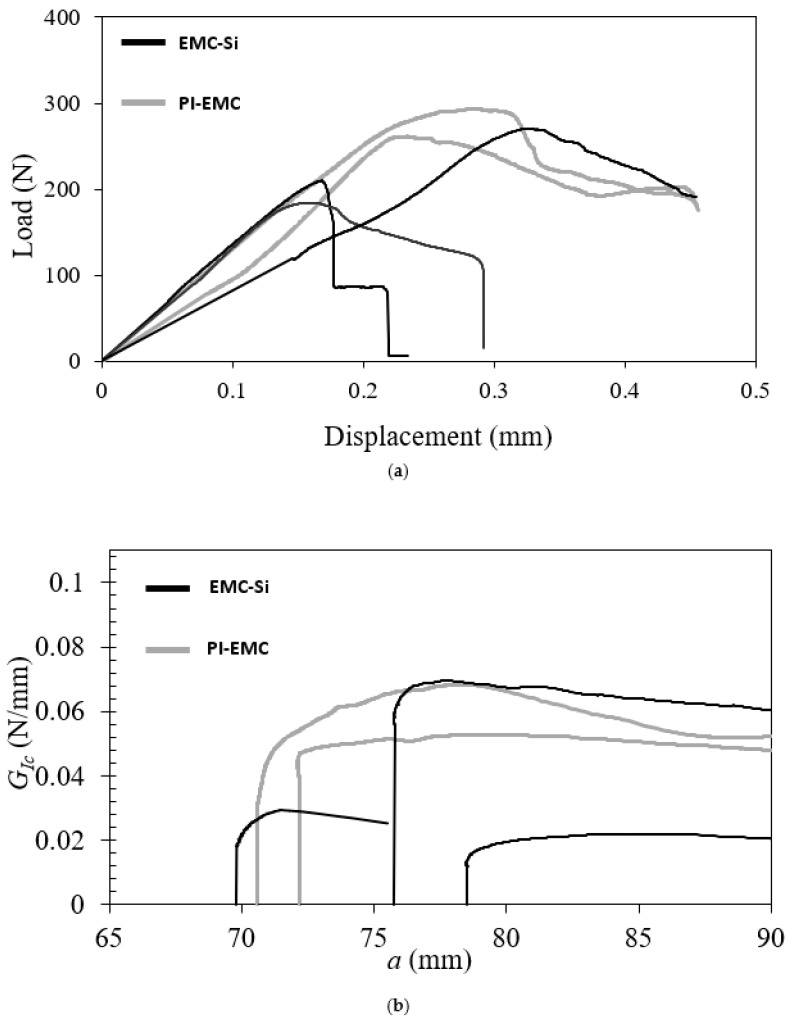
EMC–Si and PI–EMC fracture test results: Comparison of (**a**) load–displacement and (**b**) R–curves at room temperature. Similar colors/line types show the results for the same conditions.

To provide context, the comparison of fracture results at room temperature for the EMC–Si and PI–EMC interfaces will shortly be presented, as stated in [35]. Figure 2 compares the load–displacement and R–curves for the EMC–Si and PI–EMC interfaces.

The results presented in Figure 2 indicate a $G_{Ic}$ of 0.051 ± 0.001 N/mm for EMC–Si and 0.037 ± 0.024 N/mm for PI–EMC. The maximum load was 276.0 ± 22.6 N for the EMC–Si interface and 221.3 ± 44.1 N for the PI–EMC interface. For the EMC–Si interface, some scatter was observed in the results, primarily due to silicon failure at the sample edges, which required higher loads to propagate the crack. However, given that we are dealing with a multilayer sample with individual layers ranging in thickness from a few microns to even a few nanometers, the mechanical test setup, which is several orders of magnitude larger in size, can introduce variability. Any small deviation in the test setup can cause scatter in the results. PI–EMC specimens showed less scatter, although some variability was still noticeable due to differences in pre-crack lengths caused by contaminations. Despite these variations, values for $G_{Ic}$ and maximum displacement were similar between the two interfaces. During testing under these conditions, other failure mechanisms such as mixed-mode or silicon failure were also observed. Mixed-mode results indicated that stress concentrations and defects at the edges of the silicon layer caused by the dicing procedure led to the crack path deviating from the interface, resulting in a mixed-mode or silicon failure mechanism.

#### 3.1.2. Impacts of High and Low Temperatures

- Low temperature

**Figure 3:**
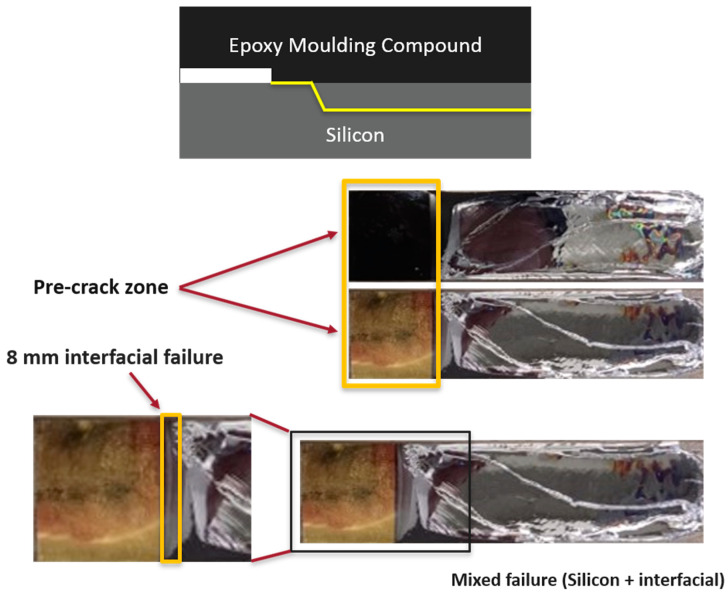
EMC–Si specimens’ fracture surface at low temperature. The yellow line presented in the scheme shows the crack propagation path after a short interfacial crack propagation—the crack kinks to the silicon layer and propagates in this layer until the joint fractures.

For low-temperature tests (−20 °C), three out of six specimens of the EMC–Si interface exhibited an interfacial failure mode. There were two phases of crack propagation: initially, an interfacial cracking with a length of 2 to 8 mm for different samples tested, attributed to the brittleness of the EMC at low temperatures, which reduces its ability to deform plastically, followed by a crack propagation through the silicon (Figure 3). Partial EMC remnants on the silicon surface suggest incomplete interfacial decohesion.

**Figure 4:**
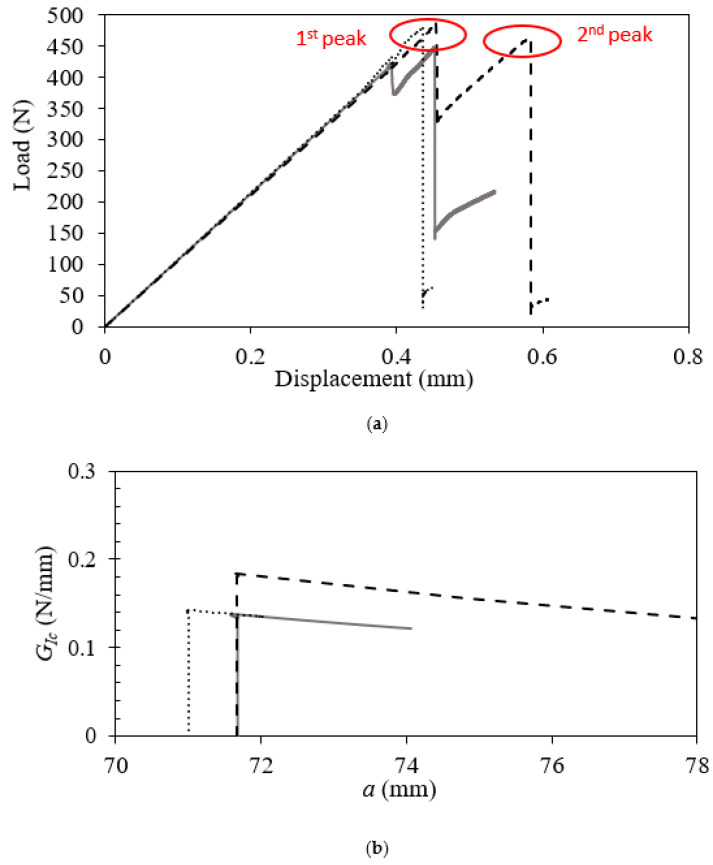
Mode I quasi-static low-temperature (**a**) load–displacement and (**b**) R–curves for the EMC–Si interface. The first peak in (**a**) indicates initial crack propagation, and the second peak indicates crack extension through the silicon substrate. Each line represents a specific sample test.

Following the CBBM approach, the load–displacement data and the R–curves were obtained, as shown in Figure 4.

The load–displacement curves reveal that the initial compliance of the three specimens is nearly identical. Regarding the maximum load, similar values were extracted at 467.0 ± 19.2 N, while the fracture energy was 0.136 ± 0.053 N/mm. It is also important to clarify that in the load–displacement curves, two peak loads are observed. The first peak corresponds to the initial step in interfacial crack propagation, while the second peak indicates the extension of the crack through the silicon substrate. Therefore, only the initial portion of the load–displacement curves was analyzed for interfacial fracture energy measurements. Similarly, the R–curves were obtained accordingly.

- High temperature

It takes nearly 15 min to reach the desired temperature. The temperature of the sample was monitored using a thermocouple during the experiments. Due to the thermal expansions of the connectors and the test setup, the load applied to the samples changes before starting the test. During the 15 min heating period, thermal gradients may arise within the sample due to differences in thermal conductivity between materials (e.g., EMC vs. silicon). These gradients could lead to uneven expansion/contraction, potentially altering interfacial stress distributions and crack initiation paths. For instance, the EMC (epoxy molding compound) may soften faster than the silicon substrate, creating localized stress concentrations at the interface. On the other hand, slow heating allows time for thermally induced residual stresses to develop, especially at bi-material interfaces with mismatched coefficients of thermal expansion (CTE). For example, EMC (CTE ≈ 60–70 ppm/°C) (CTE at *T* < $T_{g}$) and silicon (CTE ≈ 3 ppm/°C) will expand at different rates, generating interfacial shear stresses that could weaken adhesion before testing begins [43]. CTE values are manufacturer-dependent, as they are influenced by variations in base materials, additives, and processing methods. Polymers such as EMC and polyimide exhibit time-dependent behavior (e.g., stress relaxation) at elevated temperatures. The 15 min hold time may allow the partial relaxation of these materials, reducing their stiffness and fracture resistance.

**Figure 5:**
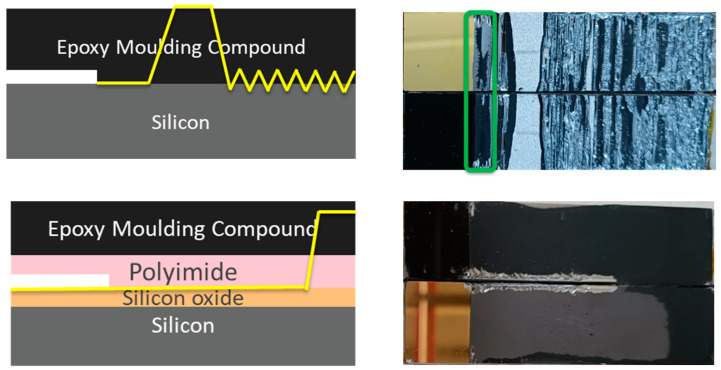
Fracture surface of EMC–Si and SiO_2_–PI at high temperature. Yellow lines show fracture path. Green line indicates the interfacial fracture area of the tested EMC–Si.

As shown in Figure 5 for the EMC–Si samples, the crack started in the pre-crack, propagated in the interface, and then kinked to the adhesive and finally returned near the interface, alternating from EMC to silicon. Despite the crack kinking to other layers, it is possible to consider only the initial part of crack propagation in the interface since it propagated across the entire width of the specimen. The ${SiO}_{2}$–PI interface was also tested under the same loading conditions, obtaining interfacial failure with all six specimens tested. It is important to note that failure in the interface was not possible to obtain at room or low temperatures.

Using modifiers, such as inorganic fillers, is effective for enhancing the overall strength, stiffness, and wear resistance of polymers (EMC and PI). Additionally, they help reduce the polymer’s coefficient of thermal expansion (CTE), thereby minimizing CTE mismatches with surrounding materials. It has been shown that the liquid maleimide-modified epoxy mold compound (EMC), which contains a SiO_2_ filler, did not exhibit a clear glass transition temperature, resulting in minimal changes in physical properties with temperature variations [44].

**Figure 6:**
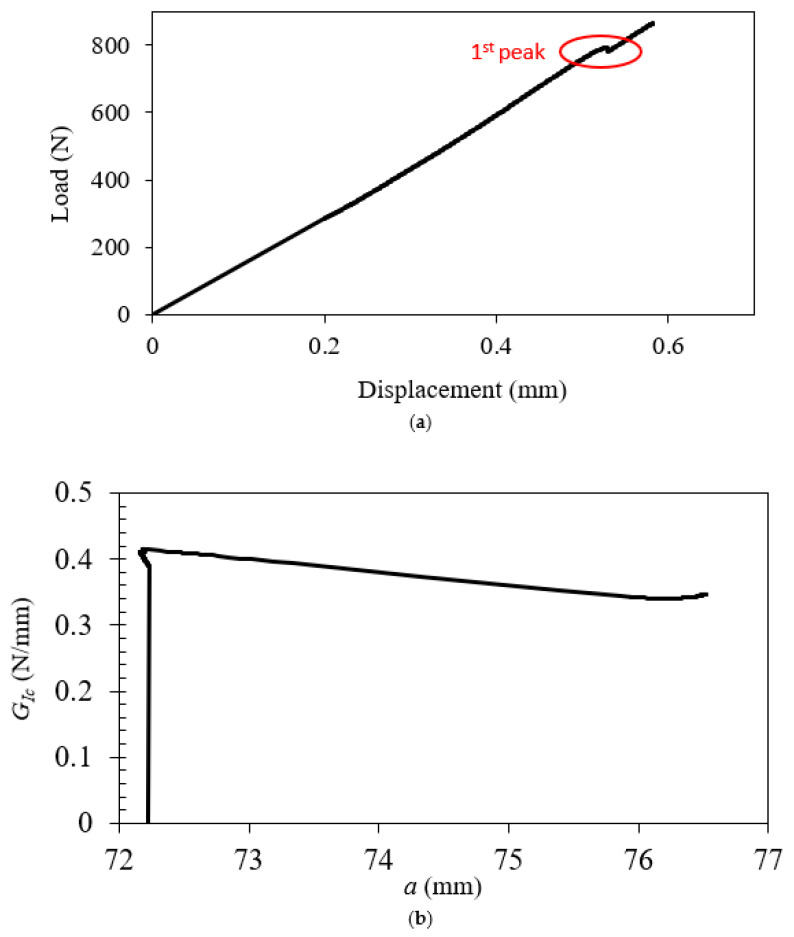
(**a**) Load–displacement and (**b**) R–curves of EMC–Si at a high temperature (100 °C). The first peak in (**a**) indicates a slight kinking of the crack into the silicon layer.

The load–displacement curve and R– curve of EMC–Si at 100 °C are depicted in Figure 6.

From this result, it is evident that the interfacial failure corresponds to a maximum load of 864 N with a fracture energy of 0.38 N/mm. An initial peak in the load–displacement curve can be detected, likely due to a small kinking of the crack to the silicon layer located at the pre-crack tip near the edges. This brittle failure may be responsible for the peak difference. Regarding the R–curve for the EMC–Si interface, a slight increase is detected at the end of the curve. This is due to the crack starting to progressively kink to the EMC and then to the adhesive layers. This crack kinking occurs due to the decrease in the mechanical properties of the adhesive at high temperatures.

**Figure 7:**
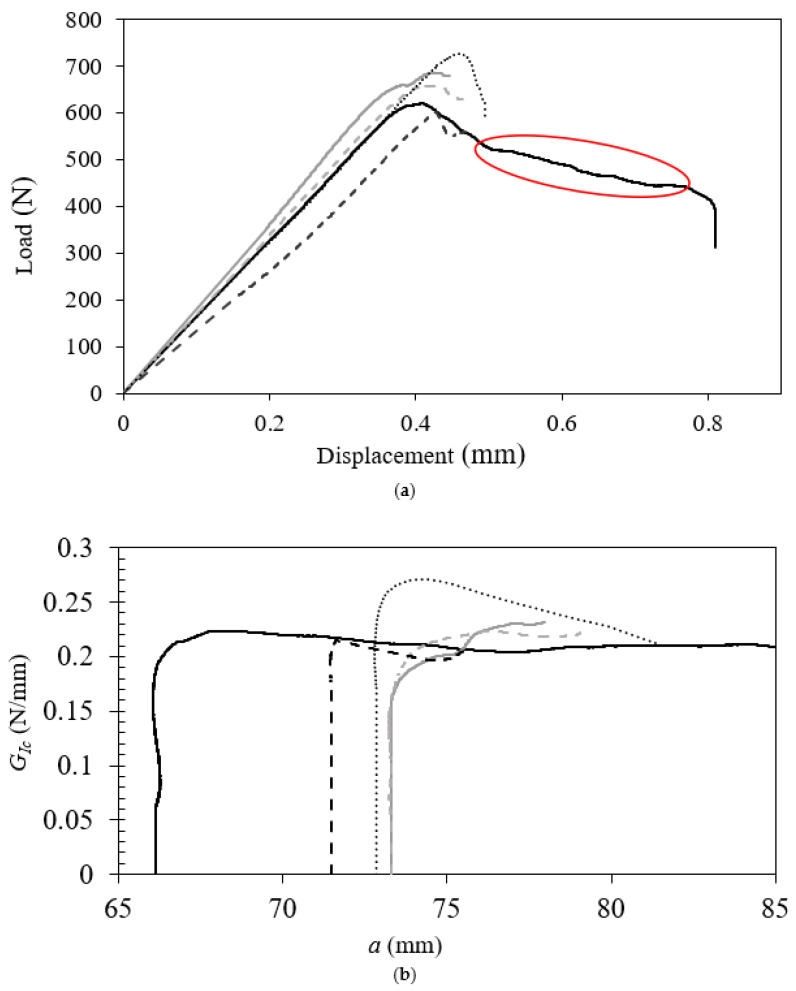
(**a**) Load–displacement and (**b**) R–curves of SiO_2_–PI at high temperature. The red circle in (**a**) indicates a change in failure mechanism. Each line in (**a**) and (**b**) represents a specific sample test result.

The load–displacement curve and R–curve of SiO_2_–PI are depicted in Figure 7. Based on these results, it can be concluded that interfacial failure occurs at a maximum load of 655.4 ± 51.9 N with a fracture energy of 0.217 ± 0.015 N/mm.

It should be noted that testing specimens at elevated temperatures degrades the properties of all materials involved, including the interfaces between them. Since different materials and interfaces exhibit varying sensitivities to temperature changes, the rate of reduction in their mechanical properties differs, leading to changes in the weakest points and failure mechanisms. Additionally, increasing the environmental temperature significantly alters the distribution of residual stresses. Moreover, due to the differing thermal expansion coefficients of the materials used in each sample, elevated temperatures induce additional stresses both at the interface and within each layer. These factors collectively contribute to changes in the failure mechanisms and mechanical properties of the interfaces tested at elevated temperatures. It has been shown that the most influential factor in interfacial delamination is the value of the coefficient of thermal expansion of the epoxy molding compound above its glass transition temperature [44]. The maximum safe value of CTE for the epoxy molding compound, ensuring that it does not exceed the critical energy release rate at the EMC–Si interface, has been identified to be 42.5628 ppm/°C.

**Figure 8:**
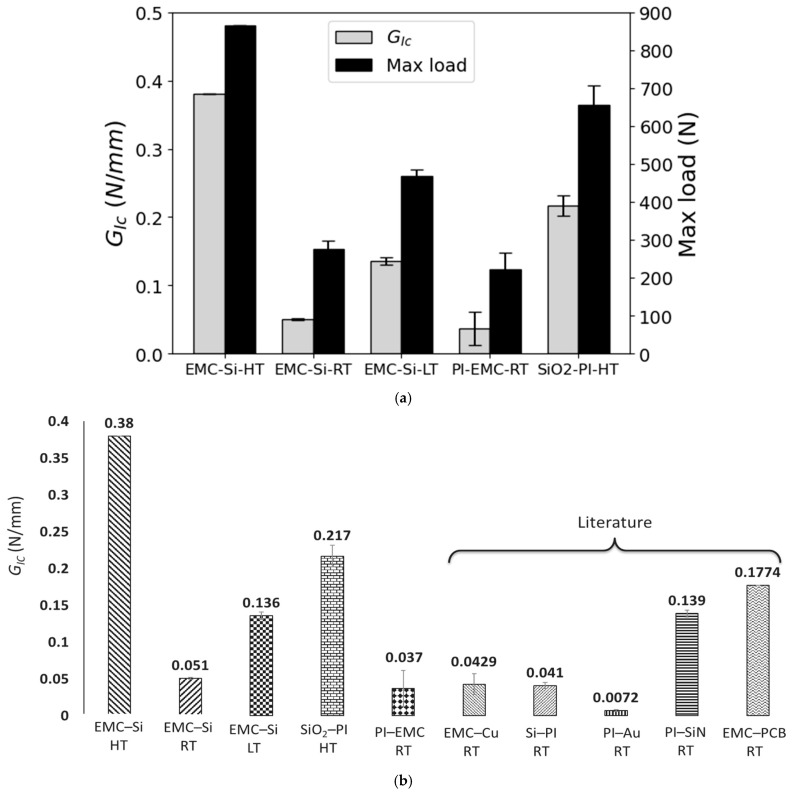
(**a**) $G_{Ic}$ comparison of quasi-static fracture tests at LT, RT, and HT, and (**b**) $G_{Ic}$ comparison of tested interfaces at different temperatures with EMC–Cu [46,47,48], PI–Si, PI–SiN, PI–Au [26], and EMC–PCB [14] interfaces found in the literature.

Figure 8a compares the mode I fracture energy and maximum failure load of different types of bi-material interfaces tested in this study.

It can be concluded that, when comparing the interfaces previously studied by Morais et al. [35] at room temperature, EMC–Si requires higher loads to delaminate compared to PI–EMC. Additionally, the fracture energy of EMC–Si is, on average, 37.8% higher than that of PI–EMC. It was found that the tested specimens in this study presented higher fracture energy values at high temperatures compared to low temperatures. Based on these results, it can be concluded that for the EMC–Si interface, the change in fracture energy is not proportional to the testing temperature. A decrease in fracture energy is observed from low to room temperatures, followed by an increase from room to high temperature. In the literature, it is common to observe a lower fracture energy at higher temperatures [45]. However, other results have also been obtained in EMC–Cu [15] for mode II, where the fracture energy increased up to the *T_g_* of the EMC. This can be due to residual stresses that may be more accentuated at lower temperatures.

In terms of the failure mechanisms observed for the EMC–Si interface, the results were relatively similar at low and high temperatures. At low temperatures, there was a small interfacial delamination followed by silicon failure, while at high temperatures, there were instances of adhesive and mixed-mode failure. At low temperatures, this failure is due to the fact that microcracks at the edges of the wafer, produced either during DCB manufacturing or during the cutting procedure of wafer manufacturing, created high-stress concentrations in the silicon layer that allowed crack propagation away from the interface. Such stress concentrations can be accentuated with temperature since the specimen is composed of materials with dissimilar coefficients of thermal expansion. At high temperatures, as explained previously, the adhesive can play a role in the kinking of the crack. When subjected to high temperatures, its mechanical properties can decrease to a point where the interface or the other materials are no longer the weakest point in the test sample, allowing a mixed failure behavior. At room temperature, these samples showed a higher rate of mixed-mode failure due to higher micro-defects detected at the edge of the silicon layers. Despite this, for all of the testing scenarios of this interface, it was possible to observe some samples with interfacial delamination.

In contrast, for the SiO_2_–PI interface, there was a change in failure mode corresponding to temperature variation. At room and low temperatures, silicon failure occurred due to factors such as the presence of micro-cracks in the silicon layer, where the interface was not the weakest link in the joint. Thermal stress induced at these temperatures also facilitated crack propagation from existing micro-cracks. However, at high temperatures, crack propagation through the interface was observed in all tested specimens. This change could be attributed to reduced interfacial properties or decreased stress concentration at the silicon layer under high-temperature conditions.

Figure 8b shows a comparison of the results obtained in this study for EMC–Si at different temperatures, PI–EMC at room temperature, and SiO_2_–PI at high temperatures with literature values for EMC–Cu [46,47,48], PI–Si, PI–Au (gold), PI–SiN [26] (silicon nitride), and EMC–PCB [14], all at room temperature. The data reveal that at room temperature, the fracture energy values are similar across different material combinations. However, at both low and high temperatures, the fracture energy values are noticeably higher. It is crucial to point out that for the EMC–PCB interface, the fracture energy decreases at higher temperatures, whereas this is not observed for EMC–Si. In a prior study [26], the PI–SiN interface was analyzed using a custom-developed micro-tester. Pre-cracks were induced using a deposited gold film and subjected to 100,000 cycles at a low level to ensure full development of the pre-crack tip. Among the conclusions drawn, it was noted that the higher toughness may be attributed to the increased roughness of the silicon nitride surface, enhancing mechanical bonding with the polyimide thin film. For EMC–Cu [48], aluminum blocks were attached to the specimens and delaminated in mode I. Regarding the EMC–PCB test [14], asymmetric double cantilever beam (ADCB) tests were performed to implement various mixed modes by varying the thickness ratios of the top and bottom metal jigs, successfully creating a finite element (FE) model that predicted the interfacial delamination, including damage initiation and crack propagation, of the bi-material interface.

### 3.2. Fatigue

During fatigue testing, achieving clear interfacial failure along the entire length of the specimen was a significant challenge. Due to the fragile behavior of silicon, the crack kinked into the silicon material in 30% of the cases after a small interfacial delamination. The remaining specimens did not show any delamination at the desired interface, and the pre-crack propagated directly to the silicon bulk material. Considering the scope of this paper, only the interfacial failure results are presented and discussed. EMC–Si and PI–EMC interfaces will be compared at room temperature from a previous study [35] conducted as part of our research, and the temperature effects at room and low temperatures will be compared for the EMC–Si interface. Table 2 shows different sample configurations for each condition.

#### 3.2.1. Room Temperature

Room temperature fatigue results at 23 °C are as follows: EMC-Si-F-RT: *m* between 5.9–8.7 for fatigue loads of 285 to 375 N, and PI-EMC-F-RT: *m* between 11–20 for fatigue loads of 150 to 340 N. While a higher *m* value is sometimes associated with a higher crack growth rate, it should be noted that *m* alone does not necessarily indicate faster or slower fatigue crack growth in materials. This parameter must be interpreted alongside the fatigue threshold energy and the material parameter (C) used in the Paris law equation. However, the results of this study show that at higher temperatures, the crack growth rate (*da*/*dN*) in the stable fatigue crack growth region (second phase of the Paris law curve) is higher compared to low- and room-temperature conditions. Since microchips typically operate at higher temperatures, these experimental data highlight the importance of characterizing wafer interfaces under elevated temperature conditions. A total of 10 specimens of the EMC–Si interface were manufactured, and three specimens showed an interfacial failure where the crack propagated initially at the desired interface and then kinked to one of the adjacent materials.

**Figure 9:**
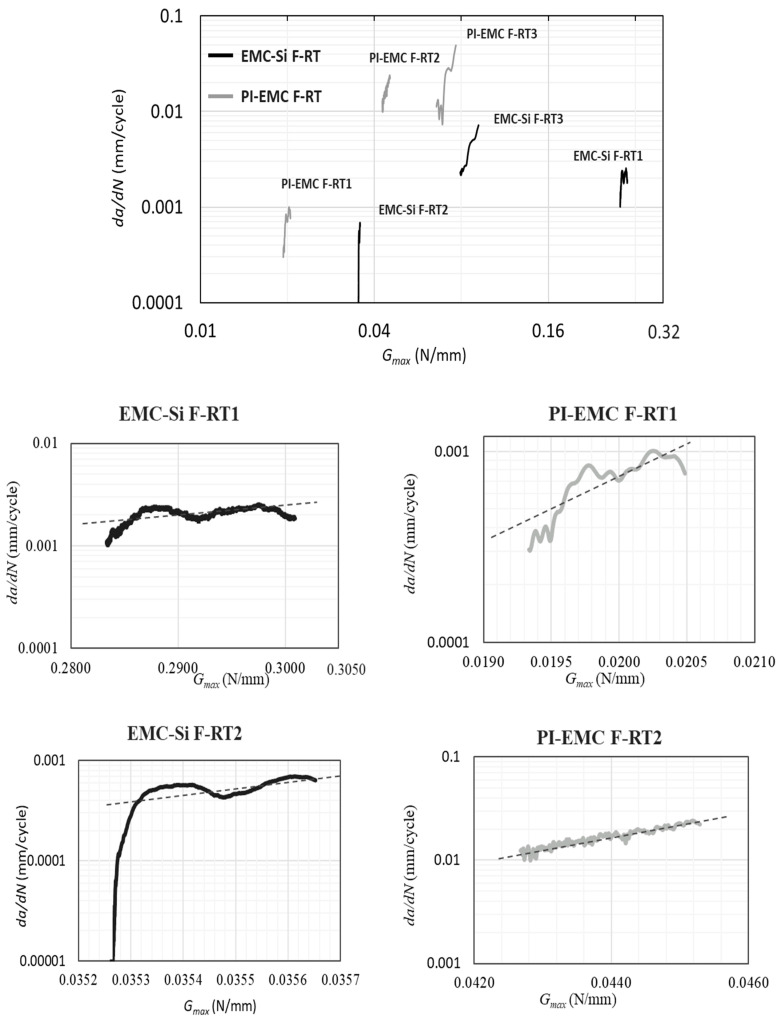

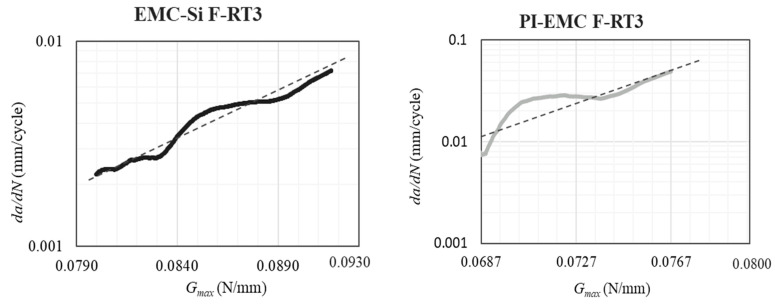
Detailed view of Paris law curves for different samples tested from both EMC–Si and PI–EMC interfaces. The dotted lines are the linear trendline of the curves.

The observed fracture mechanisms were similar to those seen in quasi-static testing under the same temperature conditions, except for the EMC–Si samples. The EMC–Si samples used in this test came from a different batch than those used for quasi-static testing at room temperature. Initially, the crack propagated along the pre-crack, but afterward, it kinked to the silicon layer, likely due to stress concentrations and microcracks present in the silicon layer. It can also be observed that the crack propagation occurs faster through the center of the specimen while its length is shorter at the edges of the specimen. The EMC–Si and PI–EMC results are represented by the Paris law curves presented in Figure 9, with the PI–EMC results obtained from Morais et al. [35].

In the PI–EMC and EMC–Si results, significant scatter among the curves is evident regarding the $G_{th}$. Determining $G_{th}$ is crucial as it indicates the minimum energy required to initiate a fatigue crack. However, providing an exact value is challenging as it necessitates reducing the maximum load until the crack growth rate reaches at least 1 × 10^−6^ mm/cycle. In load-controlled fatigue tests, reducing the maximum load decreases the obtained $G_{th}$, eventually reaching the true $G_{th}$ of the bi-material interface. The fatigue test results demonstrate a linear relationship between $G_{th}$ and the applied maximum fatigue load for the PI–EMC interface [35].

Analyzing the *m* reveals that increasing the load also slightly increases this parameter for the EMC–Si interface, while for PI–EMC, no trend was identified. However, further fatigue results with a wider range of fatigue loads are required for a more concrete conclusion.

While $G_{th}$ exhibits scatter, the *m* values are consistent across different fatigue tests conducted at various load levels. This supports the assumption that *m* values are more influenced by material parameters rather than the level of fatigue load.

#### 3.2.2. Effects of High and Low Temperatures

- Low temperature (−20 °C)

**Figure 10:**
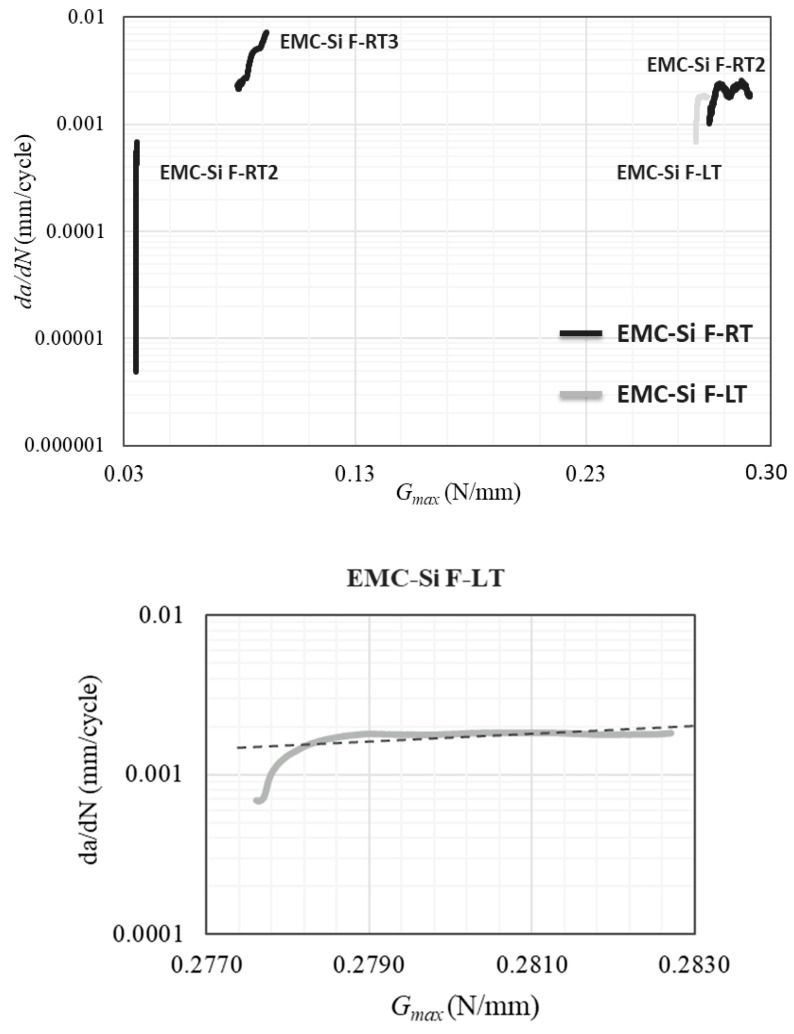
Paris law curve of the EMC–Si interface at low temperatures. The dotted line is the linear trendline of the curve.

The low temperature presented a higher challenge to successfully delaminate the specimen at the desired interface. In total, 10 specimens of the EMC–Si interface were manufactured and tested, and only one presented an interfacial fatigue failure at the desired interface. Similar to the results obtained from the quasi-static tests, some samples presented failure in silicon. As previously described, failure in silicon can occur due to the presence of microcracks at the edges of the specimen and also due to the redistribution of stresses due to a decrease in temperature. However, the percentage of interfacial failures of these samples at low temperatures decreased significantly from 50% under quasi-static conditions to 10% under fatigue conditions. One possible explanation for this reduction is that fatigue loading can alter the failure mechanism, inducing different types of stresses and crack propagation patterns compared to quasi-static loading. To draw more definitive conclusions, it is necessary to test a larger number of specimens. A larger sample size would provide more data, helping to confirm whether the observed trend is consistent and significant and allowing for a better understanding of the underlying mechanisms driving the differences in failure modes between quasi-static and fatigue conditions. Figure 10 shows the Paris law curve of the EMC–Si F–LT specimen and its comparison with the results obtained for the same interface tested at room temperature.

When comparing the effect of temperature, specimens EMC-Si-F-RT-1 and EMC-Si-F-LT are noteworthy as they experienced the same maximum load during testing. Upon analysis, it is noted that the crack growth rate shows a similar range of values, ranging from 1 × 10^−3^ to 1 × 10^−2^ mm/cycle. However, a significant decrease in the *m* value is observed with the decrease in temperature from 23 °C to −20 °C, with values dropping from 6.3 to 2.2. This means that the interface is more sensitive to the change in $G_{th}$ at room temperature than at low temperatures. Regarding the threshold energy ($G_{th}$), providing an exact value is challenging as it necessitates reducing the maximum load until the crack growth rate reaches 1 × 10^−6^ mm/cycle.

- High temperature (100 °C)

Similar to the quasi-static test at high temperatures for the SiO_2_–PI interface, interfacial delamination was also observed in all specimens tested under cyclic loading. The most common crack path observed started with the crack initiating in the pre-crack and continuing to propagate through the interface until it kinked to the adhesive or in mixed mode between the silicon and EMC.

**Figure 11:**
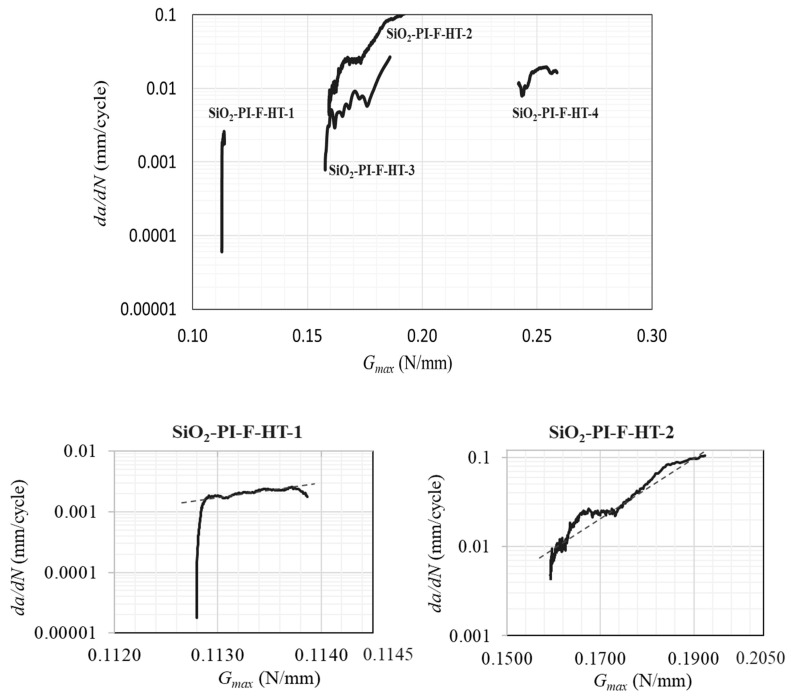

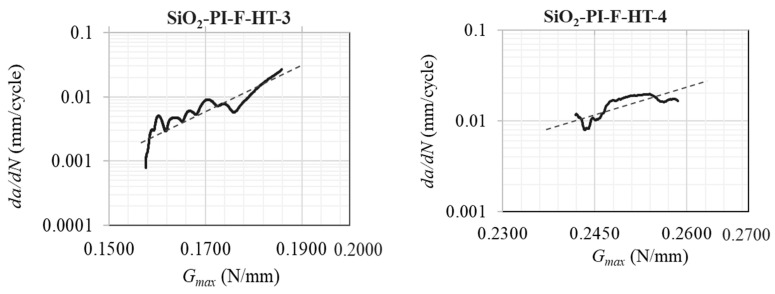
Paris law curves of the SiO_2_–PI interface. The dotted lines are the linear trendline of the curves.

As explained earlier, the crack tends to propagate through the weakest point in the specimens. However, interfacial failure occurs if the crack does not advance through microcracks that might be present in the silicon layer. If stresses concentrate around these defects, it is possible that the weakest point in the sample shifts from the interface to the defects themselves. At higher temperatures, it was found that it is possible to obtain interfacial failure contrary to what happens at room and low temperatures. It can be concluded that both quasi-static and fatigue loading produce the same failure mechanism at the same testing temperature. The Paris law is depicted in Figure 11.

Table 3 summarizes the *m* values and maximum fatigue load obtained from the tested specimens. 

**Figure 12:**
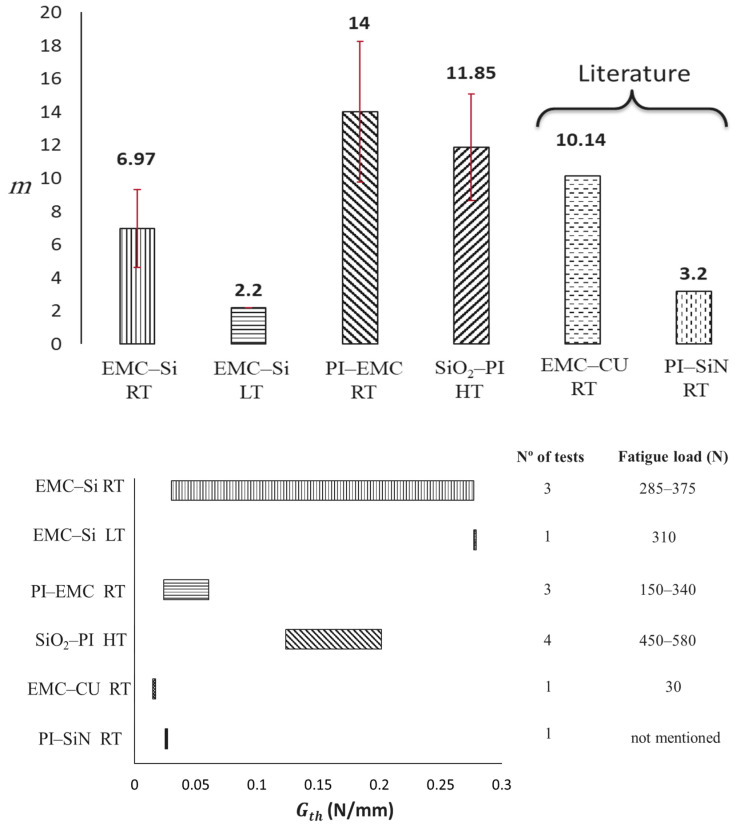
*m* comparison for interfaces and different temperatures with EMC–Cu [49] and PI–SiN [26] interfaces from the literature, and $G_{th}$ comparison for different interfaces and temperatures with EMC–Cu [49] and PI–SiN [26] interfaces from the literature.

From these results, an *m* ranging from 10.5 to 12.9 and a $G_{th}$ of 0.163 ± 0.039 N/mm were obtained. As stated before, temperature considerations for bi-material interfaces are mostly seen for high-temperature conditions. To interpret the fatigue results, they were compared with findings from the literature, as shown in Figure 12. In one of the studies [26], DCB specimens were fabricated with aluminum bars as supports and tested using a custom-developed micro-tester under mode I load-controlled fatigue conditions at room temperature. The maximum fatigue load of 30 N was applied at 0.33 Hz to the sample. The interfacial failure was observed only for a portion of the specimens, as the crack kinked to other materials after approximately 14 mm of interfacial propagation for the SiN–PI interface. The obtained results are shown in Figure 12. The EMC–Cu interface [49] has been also analyzed in another study. Aluminum blocks were used instead of a metal backing, and by using an apparatus for mixed modes, the interfacial delamination of the specimen was tested for mode I and mixed modes with a frequency of 2 Hz. It is important to note that for both studies, the Paris law curves were obtained using ∆G as the fatigue crack growth parameter instead of the $G_{max}$ considered in the current work.

Regarding the *m* values, they vary significantly according to the test temperature; however, they present values of a similar order of magnitude. In the PI–SiN study, the *m* value is lower than the one obtained for PI–EMC and SiO_2_–PI. However, it is important to note that a direct comparison of the results is not feasible due to differences in loading conditions, temperature (for SiO_2_–PI), frequency of fatigue testing, and specimen size between the two studies. The *m* value for the EMC–Si interface was lower than that of the PI–EMC interface, which were both tested at room temperature. The *m* values for both interfaces are within the range found in the literature. Generally, testing under different fatigue loading levels for the same temperature conditions did not significantly vary the *m* value.

The $G_{th}$ of the EMC–Si interface shows the highest values not only at room temperature but also at low temperatures. Thermal effects were shown to have a considerable impact on results. Even if the threshold energy was within the range obtained for room temperature (for EMC–Si), the *m* value was 168% higher at room temperature than at low temperatures. For the SiO_2_–PI interface, the $G_{th}$ of 0.163 ± 0.039 N/mm was higher than the results in the literature but within the range tested for EMC–SI in this study. It should be noted that the $G_{th}$ reported here is not the true $G_{th}$ that should be considered for a safe life design. As already discussed, $G_{th}$ is a function of the applied fatigue load and converges when the load is sufficiently low to achieve a crack growth rate of 1 × 10^−6^ mm/cycle. However, in the current study, the fatigue load was not low enough to reach such a slow rate of crack growth. Thus, when compared to the literature, these values appear higher and very scattered due to the change in maximum load. Analyzing the true fatigue threshold of such interfaces can be considered for future studies.

## 4. Conclusions

This paper investigates the quasi-static and fatigue behavior of three interfaces: EMC–Si, SiO_2_–PI, and PI–EMC, with a focus on the effects of temperature. Key findings reveal that thermal variations profoundly influence interfacial properties and failure mechanisms. For the EMC–Si interface, fracture energy increased by 180% at high temperatures (0.38 N/mm at 100 °C) compared to low temperatures (0.136 N/mm at −20 °C), driven by thermal stress redistribution and material softening. Higher temperatures degrade materials and interfaces, altering failure points and mechanisms due to thermal expansion mismatches and residual stress redistribution. Conversely, the SiO_2_–PI interface shifted from silicon-dominated failure at low temperatures to interfacial delamination at high temperatures, highlighting the temperature sensitivity of adhesion strength. Temperature affects failure mechanisms and fatigue strength, with the *m* value for EMC–Si decreasing from 6.97 ± 2.33 at room temperature to 2.2 at −20 °C. SiO_2_–PI showed interfacial delamination under fatigue at high temperatures with an *m* value of 11.85 ± 0.93. Testing under varying fatigue loads did not significantly impact the *m* value, but more tests are needed to accurately determine $G_{th}$ and fatigue thresholds, especially for the PI–EMC interface.

The results of this study have practical implications for enhancing semiconductor reliability, particularly in chip packaging. By understanding the impact of temperature on fracture behavior at bi-material interfaces, manufacturers can optimize materials and bonding techniques, thereby improving the durability of integrated circuits (ICs) under varying thermal conditions.

## Figures and Tables

**Table 1:** Material properties [36,37,38].

Material	Ultimate Tensile Strength (MPa)	Poisson’s Ratio	Young’s Modulus (GPa)
Silicon	170	0.28	130
EMC	120	0.28	24
PI	215	0.35	2.5
SiO_2_	129	0.3	75

**Table 2:** Testing conditions under quasi-static and fatigue loading (RT, LT, and HT refer to room temperature, low temperature, and high temperature, respectively. QS and F stand for quasi-static and fatigue fracture tests, respectively).

Temperature	Material Code	EMC–Si		SiO_2_–PI		PI–EMC	
		QS	F	QS	F	QS	F
High (100 °C)	SiO_2_-PI-F-HT	X	*	X	X	*	*
RT (23 °C)	EMC-Si-F-RT PI-EMC-F-RT	X	X	*	*	X	X
Low (−20 °C)	EMC-Si-F-LT	X	X	*	*	*	*

* Mixed-mode failure mechanism. X interfacial failure mechanism.

**Table 3:** High-temperature fatigue results for the SiO_2_–PI interface.

Temperature	Code	*m*	Maximum Fatigue Load
100 °C	SiO_2_-PI-F-HT	10.5–12.9	450–580 N

## Data Availability

The original contributions presented in the study are included in the article, further inquiries can be directed to the corresponding author.

## References

[1] Müller G. (2007). The Czochralski Method—Where we are 90 years after Jan Czochralski’s invention. Cryst. Res. Technol..

[2] Chen A., Lo R.H.Y. (2016). Semiconductor Packaging: Materials Interaction and Reliability.

[3] Lu D., Wong C.P. (2009). Materials for Advanced Packaging.

[4] Müller R., Heckmann K., Habermann M., Paul T., Stratmann M. (2000). New Adhesion Promoters for Copper Leadframes and Epoxy Resin. J. Adhes..

[5] Dudek R., Grossmann G., Zardini C. (2011). Popcorn Cracking. The ELFNET Book on Failure Mechanisms, Testing Methods, and Quality Issues of Lead-Free Solder Interconnects.

[6] Samet D., Kwatra A., Sitaraman S.K. A Compliance-Based Approach to Study Fatigue Crack Propagation for a Copper-Epoxy Interface. Proceedings of the InterPACK2015.

[7] Kwatra A., Samet D., Sitaraman S.K. (2015). Effect of thermal aging on cohesive zone models to study copper leadframe/mold compound interfacial delamination. Proceedings of the 2015 IEEE 65th Electronic Components and Technology Conference (ECTC).

[8] Mahan K., Han B. (2014). Four Point Bending Test for Adhesion Testing of Packaging Strictures: A Review. J. Microelectron. Packag. Soc..

[9] Krieger W.E.R., Raghavan S., Kwatra A., Sitaraman S.K. (2014). Cohesive zone experiments for copper/mold compound delamination. Proceedings of the 2014 IEEE 64th Electronic Components and Technology Conference (ECTC).

[10] Reeder J., Crews J. (1989). The mixed-mode bending method for delamination testing. Proceedings of the 30th Structures, Structural Dynamics and Materials Conference.

[11] Tay A.A.O., Phang J.S., Wong E.H., Ranjan R. A Modified Button-Shear Method for Measuring Fracture Toughness of Polymer-Metal Interfaces in IC Packages. Proceedings of the 53rd Electronic Components and Technology Conference.

[12] Shaygi M., Wunderle B., Arnold J., Pflugler N., Pufall R. (2019). Button Shear Fatigue Test: Fracture-Mechanical Interface Characterisation under Periodic Subcritical Mechanical Loading. Proceedings of the 2019 25th International Workshop on Thermal Investigations of ICs and Systems (THERMINIC).

[13] Gurumurthy C., Kramer E.J., Hui C.Y. (2006). Controlling Interfacial Interpenetration and Fracture Properties of Polyimide/Epoxy Interfaces. J. Adhes..

[14] Um H.J., Lee S.M., Lee D.W., Ha S., Kim H.S. (2023). Mixed mode fracture toughness of epoxy molding compound/printed circuit board interface of semiconductor packages with respect to temperature and moisture. Eng. Fract. Mech..

[15] Tran H.T., Shirangi M.H., Pang X., Volinsky A.A. (2014). Temperature, moisture and mode-mixity effects on copper leadframe/EMC interfacial fracture toughness. Int. J. Fract..

[16] Poshtan E.A., Rzepka S., Silber C., Wunderle B. (2015). Accelerated determination of interfacial fracture toughness in microelectronic packages under cyclic loading. Proceedings of the 2015 IEEE 65th Electronic Components and Technology Conference (ECTC).

[17] Asmah M.T. (2016). Study of polyimide coated chip and mold compound adhesion. Proceedings of the 2016 IEEE 37th International Electronics Manufacturing Technology (IEMT) & 18th Electronics Materials and Packaging (EMAP) Conference.

[18] Ferreira R.A., Akhavan-Safar A., Carbas R.J.C., Marques E.A.S., Karunamurthy B., da Silva L.F.M. (2024). Advancements in mechanical characterization techniques and environmental effects on bi-material interfaces in microelectronics: A literature review. J. Adhes..

[19] Sankarasubramanian S., Cruz J., Yazzie K., Sundar V., Subramanian V., Alazar T., Yagnamurthy S., Cetegen E., McCoy D., Malatkar P. (2017). High-Temperature Interfacial Adhesion Strength Measurement in Electronic Packaging Using the Double Cantilever Beam Method. J. Electron. Packag..

[20] Yeh S.S., Lin P.Y., Hsu C.K., Lin Y.S., Wang J.H., Lai P.C., Liao L.L., Lee T.Y., Chen C.H., Yew M.C. (2020). Analysis of Underfill-Polymer Interfacial Adhesive Strength by Combined Experimental and Modeling Approaches. Proceedings of the 2020 15th International Microsystems, Packaging, Assembly and Circuits Technology Conference (IMPACT).

[21] Xiao A., De Vreugd J., Pape H., Wunderle B., Jansen K.M., Ernst L.J. Establishing fracture properties of emc-copper interfaces in the visco-elastic temperature region. Proceedings of the 2009 59th Electronic Components and Technology Conference.

[22] Luo S., Wong C.P. (2005). Influence of temperature and humidity on adhesion of underfills for flip chip packaging. IEEE Trans. Comp. Packag. Technol..

[23] Lebbai M., Kim J.K., Yuen M.M.F. (2003). Effects of moisture and elevated temperature on reliability of interfacial adhesion in plastic packages. J. Electron. Mater..

[24] Poshtan E.A., Rzepka S., Silber C., Wunderle B. An in-situ numerical–experimental approach for fatigue delamination characterization in microelectronic packages. Proceedings of the 16th International Conference on Thermal, Mechanical and Multi-Physics Simulation and Experiments in Microelectronics and Microsystems.

[25] Samet D., Trilochan Rambhatla V.N.N., Kwatra A., Sitaraman S.K. (2017). A fatigue crack propagation model with resistance curve effects for an epoxy/copper interface. Eng. Fract. Mech..

[26] Zhu S.W., Shih C.P., Chiu T.C., Shen G.S. Delamination fracture characteristics for polyimide-related interfaces under fatigue loadings. Proceedings of the 2010 5th International Microsystems Packaging Assembly and Circuits Technology Conference.

[27] Dey K., Gobetti A., Ramorino G. (2023). Advances in understanding of multiple factors affecting vibration weld strength of thermoplastic polymers. J. Adv. Join. Process..

[28] Jongbloed B., Teuwen J., Villegas I.F. (2023). On the use of a rounded sonotrode for the welding of thermoplastic composites. J. Adv. Join. Process..

[29] Melese K.G., Singh I. (2023). Adhesive behavior of sisal and jute composite exposed to three months cyclic temperature variation. J. Adv. Join. Process..

[30] Rahim M.K., Suhling J.C., Jaeger R.C., Lall P. (2005). Fundamentals of delamination initiation and growth in flip chip assemblies. Proceedings of the Electronic Components and Technology, 2005. ECTC ’05.

[31] Shimamoto K., Sekiguchi Y., Sato C. (2016). The Critical Energy Release Rate of Welded Joints Between Fiber-Reinforced Thermoplastics and Metals When Thermal Residual Stress is Considered. J. Adhes..

[32] Schlottig G., Pape H., Wunderle B., Ernst L.J. (2009). Induced delamination of silicon-molding compound interfaces. Proceedings of the EuroSimE 2009—10th International Conference on Thermal, Mechanical and Multi-Physics Simulation and Experiments in Microelectronics and Microsystems.

[33] Schlottig G., Pape H., Xiao A., Wunderle B., Ernst L. (2009). How to fabricate specimens for silicon-to-molding compound interface adhesion measurements. Proceedings of the 2009 11th Electronics Packaging Technology Conference.

[34] Schlottig G., Xiao A., Pape H., Wunderle B., Ernst L.J. (2010). Interfacial strength of Silicon-to-Molding Compound changes with thermal residual stress. Proceedings of the 2010 11th International Thermal, Mechanical & Multi-Physics Simulation, and Experiments in Microelectronics and Microsystems (EuroSimE).

[35] Morais P., Akhavan-Safar A., Carbas R.J.C., Marques E.A.S., Karunamurthy B., da Silva L.F.M. (2024). Mode I Fatigue and Fracture Assessment of Polyimide–Epoxy and Silicon–Epoxy Interfaces in Chip-Package Components. Polymers.

[36] Zhang S., Mori S., Sakane M., Nagasawa T., Kobayashi K. (2012). Tensile Properties and Viscoelastic Model of a Polyimide Film. J. Solid Mech. Mater. Eng..

[37] A Background to Silicon Its Applications A.Z.o.M. https://www.azom.com/properties.aspx?ArticleID=599.

[38] Silica—Silicon Dioxide (SiO_2_), A.Z.o.M. Published 13 December 2001. https://www.azom.com/article.aspx?ArticleID=1114.

[39] (2012). Standard Test Method for Fracture Strength in Cleavage of Adhesives in Bonded Metal Joints.

[40] Yin H., Liu J., Xia H., Guo L., Ao X., Luo J., Yang Y. (2024). Effect of combination of microstructure and surface treatment on shear strength of precision bonded joints. J. Adhes..

[41] Wirries J., Vallée T., Rütters M. (2023). How to predict residual stresses of curing adhesives ab initio solely using extended rheometry. J. Adhes..

[42] Da Silva L.F.M., Öchsner A. (2008). Modeling of Adhesively Bonded Joints.

[43] Chun H., Park S.Y., Park S.J., Kim Y.J. (2020). Preparation of low-CTE composite using new alkoxysilyl-functionalized bisphenol A novolac epoxy and its CTE enhancement mechanism. Polymer.

[44] Conversion A., Ubando A.T., Gonzaga J. (2023). Interfacial Delamination Validation on Fan-Out Wafer-Level Package Using Finite Element Method. Solid State Phenom..

[45] Santos D., Akhavan-Safar A., Carbas R.J.C., Marques E.A.S., Wenig S., da Silva L.F.M. (2024). Mode I fatigue threshold energy assessment of a polyurethane adhesive: Effects of temperature and Paris law relation. J. Adhes..

[46] Trilochan Rambhatla V.N.N., Sitaraman S.K. (2023). Crowbar Loading—A New Test Technique to Characterize Interfacial Delamination. Eng. Fract. Mech..

[47] Calabretta M., Sitta A., Oliveri S.M., Sequenzia G. (2022). Copper to resin adhesion characterization for power electronics application: Fracture toughness and cohesive zone analysis. Eng. Fract. Mech..

[48] Krieger W.E.R., Raghavan S., Sitaraman S.K. (2016). Experiments for Obtaining Cohesive-Zone Parameters for Copper-Mold Compound Interfacial Delamination. IEEE Trans. Compon. Packag. Manuf. Technol..

[49] Chen Y.J., Deng Y.A., Chiu T.C. Fatigue Crack Growth on the Interface of Copper and Epoxy Molding Compound under Mixed-Mode Loading. Proceedings of the 2018 13th International Microsystems, Packaging, Assembly and Circuits Technology Conference (IMPACT).

